# Physical Exercise Counteracts Aging-Associated White Matter Demyelination Causing Cognitive Decline

**DOI:** 10.14336/AD.2024.0216

**Published:** 2024-10-01

**Authors:** Tanya H. Butt, Makoto Tobiume, Diane B. Re, Shingo Kariya

**Affiliations:** ^1^Department of Environmental Health Sciences, Mailman School of Public Health, Columbia University, New York, NY, USA.; ^2^Unit for Respiratory System & Dementia in the Division of Internal Medicine, Katsuren Hospital, Itoman, Okinawa, Japan.; ^3^NIEHS Center for Environmental Health Sciences in Northern Manhattan, Columbia University, New York, NY, USA.; ^4^Center for Motor Neuron Biology and Disease, Columbia University, New York, NY, USA.; ^5^Unit for Nervous System & Dementia in the Division of Internal Medicine, Katsuren Hospital, Itoman, Okinawa, Japan.

**Keywords:** Alzheimer’s disease;, aging, white matter demyelination, cognitive decline, physical exercise, oligodendrocyte

## Abstract

In the central nervous system, oligodendrocytes wrap around neuronal axons to form myelin, an insulating layer or sheath that allows for the efficient conductance of action potentials. In addition to structural insulation, myelin provides encased axons with nutrient, metabolic and defensive support. Demyelination, or myelin loss, can therefore cause axonal dysfunction, leading to neurological impairment and disease. In Alzheimer’s disease (AD), progressive white matter demyelination is acknowledged as one of the earliest pathologies preceding symptom onset. Unfortunately, current pharmacotherapy for slowing demyelination or promoting remyelination in AD is nonexistent. Exercise is recognized for its wide-ranging benefits to human health, including improved mental health and the prevention of lifestyle-related diseases. Mounting evidence suggests the contribution of physical activity in delaying the progression of dementia in elderly populations. Recent mechanistic studies have shown that exercise facilitates myelination in the brain through the vitalization of intrinsic pro-myelination cues, such as increased neurotrophic factors and electrical activity. In this review, we summarize and discuss the potential of physical exercise on counteracting aging-associated white matter demyelination, which causes cognitive decline in AD. We highlight the need of further basic and clinical research investigations on this topic to establish novel approaches for healthy and improved brain aging.

## Introduction

Aging is characterized by the gradual and progressive deterioration of physiological functions over a lifetime and often results in the waning of daily activity at the physical and cognitive levels. As elderly populations continue to grow, the number of people suffering from age-related health problems (e.g., muscle weakness, joint pain, hearing loss, impaired vision, depression, cognitive decline) is increasing worldwide [1]. Consequently, identifying and establishing ways for healthy and better aging is a major public interest with critical socioeconomic implications. In most countries, senile dementia (SD) is the costliest age-related condition and expenses are estimated to increase over time [2, 3]. About 80% of SD cases consist of two major disorders: Alzheimer’s disease (AD) and vascular dementia (VaD) [4]. While AD and VaD are believed to have different etiologies, both conditions disrupt cognitive functioning and increase the risk of secondary incidents and complications, including falls/traumatic injuries, dehydration/malnutrition, and infection. Many patients affected by SD also become weak and bedridden and require the expertise and assistance of multiple specialists and caregivers.

Several types of pharmacological agents are currently available to treat, but not cure, patients with dementia. For AD, the U.S. Food and Drug Administration (FDA) has approved medications that fall into two categories: drugs that mitigate cognitive symptoms by modulating synaptic transmission impaired as a consequence of the disease process, and drugs that slow the disease progression by interfering with a pathogenic peptide produced upstream of the AD neurodegenerative cascade. The first AD drug category consists of cholinesterase inhibitors (rivastigmine, donepezil, galantamine) that promote cholinergic neurotransmission and a N-methyl-D-aspartate receptor antagonist (memantine) that inhibits glutamatergic signaling. All are in clinical use globally, but only alleviate symptoms temporarily and none can halt AD-associated brain damage [5]. The second AD drug category, which recently received accelerated approval, aims to remove amyloid-beta, a pathogenic peptide widely accepted to be a hallmark of AD brain pathology. Treatments using anti-amyloid antibodies (aducanumab, lecanemab) are currently restricted to patients in the early stages of AD with mild cognitive impairment and their effectiveness has yet to be evaluated [6, 7]. Conversely, there is no FDA approved drug for the treatment of VaD. Several AD drugs, such as cholinesterase inhibitors and memantine, were evaluated in clinical trials of VaD, but only marginal positive outcomes were obtained [8-11]. Altogether, the current situation of pharmacotherapy for SD remains unsatisfactory and requires further development.

Physical exercise (PE), often simply expressed as “exercise”, is a planned, structured and repetitive type of bodily movement with the purpose of improving or maintaining one or more components of physical fitness [12]. It is a non-invasive, low-cost, personalized, and most importantly, a physiologically natural way of gaining wellness. Mounting evidence indicates the positive effect of PE as a non-pharmacological approach to prevent cognitive decline in elderly populations [13-16]. Major mechanisms proposed to account for this effect are the following [17, 18]: first, daily exercise helps maintain cerebral blood flow, which is indispensable for healthy brain function; second, it mitigates comorbidities (e.g., hypertension, hyperlipidemia, diabetes, obesity, atherosclerosis), which can increase the risk of poor cerebral blood flow; third, it stimulates the expression of neurotrophic factors, such as insulin-like growth factor 1 (IGF-1), brain-derived neurotrophic factor (BDNF), vascular endothelial growth factor (VEGF) and fibroblast growth factors (FGFs) including FGF2; fourth, it promotes clearance of amyloid-beta via glymphatic and meningeal lymphatic systems [19, 20]; and fifth, it suppresses activation of neurotoxic inflammatory molecules (e.g., tumor necrosis factor-alpha, interleukin-6, and interleukin-1beta) [21-23].

In the central nervous system (CNS), oligodendrocytes (OLs) wrap around neuronal axons and form myelin, an insulating layer or sheath that allows for the efficient axonal conductance of action potentials. Myelin also provides the ensheathed axons with nutrient, metabolic and defensive support [24, 25]. Myelin loss, namely demyelination, can cause axonal dysfunction leading to neurological symptoms. The most common demyelinating disorder that affects the CNS is multiple sclerosis (MS) [26]. Other demyelinating diseases include relatively rare disorders, such as myelin OL glycoprotein antibody-associated diseases [27], central pontine myelinolysis [28] and leukodystrophy [29]. While AD has long been considered a disease with primary atrophy of neuronal cell bodies in gray matter, growing evidence indicates early axonal demyelination in the white matter (WM) of preclinical AD brains [30-33]. Thus, slowing demyelination or promoting remyelination of the brain could represent some early therapeutic strategies for AD, before the occurrence of significant neurodegeneration. While pharmacotherapies targeting myelination have not been established yet, recent studies have demonstrated that PE can help myelinate brains [15, 34-36], suggesting a novel PE-based mechanism for counteracting cognitive decline in AD. In support of this view, some reports have indicated the therapeutic potential of PE to promote remyelination in demyelinating disorders, such as MS and central pontine myelinolysis [37-40].

In this review, we summarized, with particular focus on the association with AD pathogenesis, our latest understanding of the relationship between PE and WM demyelination. We discussed the potential of PE for preventing and slowing progression of aging-associated WM demyelination causing cognitive decline in AD.

### Evidence of early white matter demyelination in AD

Along with increased accessibility to magnetic resonance imaging (MRI) and advances in neuroimaging techniques over the past two decades, a growing number of studies report that WM hyperintensity (WMH), visualized as increased signal on T2-weighted cerebral images, is an early AD pathological feature preceding symptom onset [30, 31]. The first clear evidence of this was based on a large-scale survey study conducted on a prospective population-based cohort from the Netherlands [32], which concluded that asymptomatic elderly people with WMH have a higher risk for developing AD than those without such lesions. Another study comprising participants from the Dominantly Inherited Alzheimer Network revealed that carriers of mutations causative of AD have greater lesion volumes of WMH than their non-carrier, first-degree relatives, and that these lesions increase approximately 6 years prior to expected clinical onset [33]. This study also found that, in the preclinical stage, the severity of WMH in the mutation carriers correlated with cerebrospinal fluid (CSF) amyloid-beta levels, suggesting a mechanistic relationship between these two pathological markers.

An analysis of postmortem AD brains revealed that the accumulation of WMH is associated with myelin pallor and axonal demyelination [41-43]. Biochemical studies on WM specimens from AD patients demonstrated that CNS myelin-related components, such as myelin basic protein (MBP) and cholesterol, are significantly decreased compared to individuals without dementia [44, 45]. Longitudinal monitoring of brain MRI and histology in a Tg2576 AD mouse model revealed that demyelination precedes the detection of WMH [46]. In another AD mouse model, the 5XFAD mouse, decreased expression of MBP was measured in all memory-associated brain regions prior to emergence of amyloid-beta deposition [47, 48]. In this mouse model, treatment with neutralizing antibodies against LINGO-1, a negative regulator for myelin formation, promoted remyelination and attenuated memory deficits, suggesting involvement of myelin damage in the appearance of cognitive symptoms. Collectively, evidence from human and animal studies indicates that WM demyelination associated with AD appears preclinically.

### Molecular and cellular mechanisms underlying white matter demyelination in AD

Apolipoprotein E (apoE), which in the CNS is primarily produced by astrocytes, functions as a key regulator of lipoprotein metabolism and exists in humans as three common isoforms, E2, E3 and E4 [49]. Among these, apoE4 is recognized as the strongest genetic risk for AD [50-52] and it is known to cause aberrant lipoprotein homeostasis including reduced cholesterol efflux and biosynthesis [53]. In recent studies, apoE4 has been shown to impair CNS myelination via cholesterol dysregulation in OLs. McKenzie et al. demonstrated that OL-enriched gene expression networks generated from human postmortem brain samples are strongly dysregulated in AD [54]. Blanchard et al. analyzed postmortem brains from apoE4 carriers versus non-carriers and found abnormally deposited cholesterol within differentiating OLs of the apoE4 brains, concomitant with reduced myelination [55]. They have also demonstrated that pharmacologically facilitating cholesterol transport promotes axonal myelination and improves cognitive functions in transgenic apoE4 mice. Another study by Mok et al. demonstrated abnormal accumulation of OL precursors in the brains of apoE4 mice, suggesting a disruption of OL differentiation [56]. Using cell cultures, they also showed that apoE4 interrupts delivery of astrocyte-derived lipids to OLs, thereby impairing OL differentiation. Cheng et al. also reported the significant reduction of mature OLs in both apoE4-carrying human and transgenic mice [57]. In summary, these findings indicate that the WM demyelination in AD is associated with impaired astrocyte-to-OL transport of cholesterol, which is critical for OL precursor maturation and required for myelination. Glycolytic deficiency in OLs was also reported as a potential trigger for AD-related WM demyelination. Saito et al. analyzed RNA-seq data obtained from postmortem brains and found that the glycolytic pathway is significantly impaired in AD OLs [58]. Some mechanistic studies performed by Zhang and collaborators have indicated that, in mature OLs of AD mice, an abnormally hyperactivated mitochondrial fission enzyme dynamin-related protein 1 (Drp1) inhibits hexokinase-1, a mitochondrial enzyme that initiates glycolysis, resulting in an energy deficiency and the impairment of energy-consuming myelination processes [59]. They also demonstrated that heterozygous knockout of Drp1 in mature OLs mitigates myelin loss, axonal degeneration, and cognitive defects in 5XFAD mice. Noteworthily, this study clearly showed that mature OLs are also disabled in AD. Altogether, it appears that, in AD, OLs are impaired in both their differentiating and mature states, inevitably leading to deficits in both *de novo* myelin formation (i.e., remyelination) as well as myelin maintenance.

The relationships between such OL impairments and the two most prominent AD hallmarks, namely amyloid-beta plaques, and neurofibrillary tangles, have also been investigated. Amyloid plaques are extracellular aggregates consisting of misfolded amyloid-beta peptides, originally formed by two consecutive secretase enzymes cleaving the membrane-anchored amyloid precursor protein (APP) [60]. Depp et al. demonstrated that production of APP fragments is surged locally within the segments of swollen axons ensheathed by dysfunctional myelin and damaging myelin results in increased plaque deposition [61]. On the other hand, through a bioinformatics analysis of biopsied brain tissues from living individuals in their early AD stages, Gazestani et al. discovered a significant upregulation of genes associated with amyloid-beta production and processing in OLs, indicating their possible contribution as amyloid plaque-generating cells in AD [62]. While the origin of amyloid plaques is still under debate, these studies indicate that OL/myelin aberration is an upstream event driving amyloid-beta accumulation; this idea is consistent with the fact that WM demyelination is an early AD pathological feature preceding amyloid-beta deposition [30, 47]. In agreement, several groups have reported the potential of the pro-myelinating agent clemastine in reducing CNS amyloid pathologies and ameliorating cognitive deficits in AD mouse models [63-65]. Regarding the Drp1 hyperactivation provoking mitochondrial dysfunctions of OLs in AD [59], it is presumably not a cause but rather a consequence of amyloid-beta accumulation in these cells [66, 67]. Zhang et al. have shown that Drp1 is aberrantly hyperactivated in mature OLs cultured with cell-internalizable amyloid-beta [59, 68]. Using co-immunoprecipitation experiments, the Reddy laboratory revealed that Drp1 directly interacts with amyloid-beta whose levels are increased within the CNS cells of AD patients and mouse models. They further demonstrated that this abnormal interaction, which mediates the Drp1 hyperactivation causing excessive fission and functional defects of mitochondria, increases as disease progresses [69, 70].

On the other hand, phosphorylated tau, which forms intracellular neurofibrillary tangles upon aggregation [60], may have unique ways of spreading among CNS cells. When Ferrer et al. performed unilateral inoculation of AD patient’s brain homogenates into the mouse corpus callosum, the largest brain WM structure, they detected tau seeding and spreading in both the ipsilateral and contralateral sides [71]. This phenomenon known as “cell-to-cell transfer of tau aggregates” is recognized as one of the mechanisms involved in disease progression of different tauopathies including AD [72, 73]. In the above-described study, the phosphorylated tau deposits that appeared in the WM were exclusively detected in OLs, suggesting a central role of OLs in the progression of WM tau pathologies [71]. The tight connection between OLs and tau in AD pathogenesis is also supported by a recent study that analyzed extensive, well-characterized clinicopathological datasets of AD patients [74]. Importantly, amyloid-beta was reported to accelerate tau phosphorylation [75, 76], suggesting that tau pathologies could be a consequence of amyloid-beta accumulation in AD. This hypothesis agrees with the observations that clearance of amyloid-beta using specific antibodies lessens tau pathologies in a transgenic AD mouse model expressing both mutant APP and Tau [77], or conversely that amyloid burdens in mutant tau mice lead to enhanced tau pathologies [78, 79].

**Figure 1.**
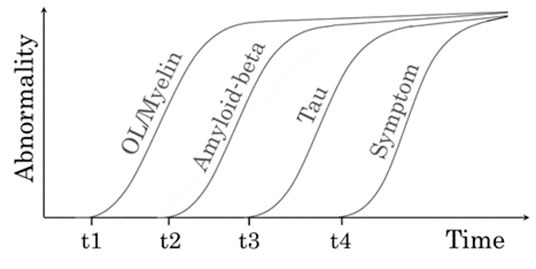
Hypothetical scheme expressing time course of biomarker abnormalities in AD. The appearance of amyloid-beta deposition (t2), which is followed by tau aggregate formation (t3), is >20 years before the AD symptom onset (t4). The emergence of WMH (t1) reflecting OL/myelin damage is detectable prior to the appearance of amyloid-beta deposition.

Most previous review articles describe the time course of AD pathological abnormalities to start with amyloid burden build-up [80-83]. As detailed, OL dysfunction and its associated WM demyelination appear to be the earliest pathogenic events detected in AD; evidence also supports that they could then lead to amyloid plaque development and subsequently to tau pathology (Figure 1). We therefore propose that OL/myelin damages or WMH could be considered among the earliest AD biomarkers, preceding the amyloid pathologies (Fig. 1). It is also important to note that approximately 30% of patients with AD are non-apoE4 carriers [84] and that regardless of one’s apoE subtype, abilities of OLs to form and maintain healthy myelin sheaths eventually decline along with natural aging [85]. Cellular senescence of OL precursors is known to reduce remyelination efficiency with increasing age [86, 87]. Consequently, we hypothesize that natural aging itself, without apoE-related but potentially in combination with other anti-myelination (or pro-demyelination) factors, can also cause OL dysfunction sufficient for the development of AD. Such an example of alternative risk factors in aged individuals is the occurrence of cerebral WM ischemic events, which can damage OLs and cause axonal demyelination [88, 89]. Ischemic WM lesions also frequently co-exist with AD pathologies [90].

### Therapeutic potential of physical exercise on white matter demyelination in AD

In APP/PS1 AD mice, enhancing myelin renewal via genetic or pharmacological interventions improved performance in memory-related tasks [65]. In 5XFAD mice, inhibition of anti-myelinating protein LINGO-1 promoted remyelination and attenuated memory deficits [47]. These results indicate the potential of enhancing myelination as a therapeutic strategy to improve AD-related cognitive decline. To date, scores of pro-myelinating molecules have been identified and various types of candidate drugs are currently under clinical trials in patients with MS [91]. Among them, clemastine has shown promising results [92], yet there is still a long way to go before its clinical application for WM demyelination in patients with AD.

**Figure 2.**
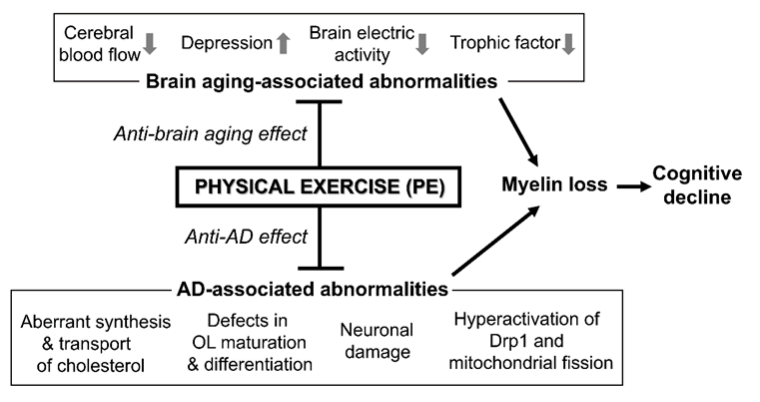
Effects of PE on myelin loss causing cognitive decline. PE can improve a number of brain aging-associated myelin loss-inducing abnormalities, such as low production and secretion of trophic factors, poor cerebral blood flow due to sedentary lifestyle and lifestyle-related cardiovascular problems, depression associated with feeling, such as loneliness and health anxiety, and low brain electric activity due to reduced activities of daily life. In addition to such anti-brain aging effects, PE can also counteract some AD-associated myelin loss-inducing abnormalities, such as aberrant synthesis & transport of cholesterol in OLs and defects in OL maturation & differentiation. Other AD-associated abnormalities that negatively affect healthy myelin formation around neuronal axons but can be counteracted by PE include mitochondrial abnormalities appearing in the CNS cells, such as OLs and neurons, and neuronal damage.

A non-pharmacological approach with a faster translational potential could be PE, whose positive effect on promoting CNS myelination is supported by mounting evidence [15, 34-36, 93, 94, 95]. The mechanisms underlying the pro-myelination effect of PE can be classified into two main categories described in Figure 2: the moderation of general aging-associated abnormalities (anti-brain aging effect) and the counteraction of more specific, AD-related abnormalities (anti-AD effect). In this last section, we review and discuss these mechanisms and the optimal PE form for moderating AD-associated demyelination.

## 1. Anti-brain aging effect

PE increases cerebral blood flow, which is indispensable for maintaining normal brain function and enabling timely myelination upon physiological requirements. PE also contributes to the prevention of lifestyle-associated diseases (e.g., cardiovascular problems), which cause poor cerebral blood flow. These first two mechanisms relating to cerebral blood flow have been well-established and are generally accepted; cerebral blood flow supplies various factors essential for the survival and functioning of all CNS cells.

The third mechanism involves pro-myelination factors and is being actively investigated. In response to PE, peripheral tissues were shown to produce and release into the bloodstream various molecules that can cross the blood-brain barrier and affect CNS cells [96]. Among them, BDNF promotes myelin formation through its action on the tropomyosin-related kinase receptor B of OL lineage cells [97-99]. While skeletal muscle-resident myocytes are well known to secrete BDNF during sustained muscle contraction [96], PE also triggers BDNF secretion directly in the CNS from astrocytes [100]. So far, the respective contribution of myocytes versus astrocytes to PE-induced total brain BDNF increase remains unclear [101]. Future studies are warranted to increase our understanding of the precise signaling pathways through which PE triggers astrocytic BDNF production. Nonetheless, the previous demonstration of a negative correlation between serum BDNF levels and future occurrence of AD suggests the importance of peripherally produced BDNF in protecting the brain from AD [102]. IGF-1, which is primarily released from the liver and then enters the CNS [103], is another exercise-induced factor capable of promoting myelination by binding to its receptor expressed in OLs [104-106]. The production of IGF-1 is induced by growth hormone, which is promptly secreted upon PE by the pituitary gland into the bloodstream [107]. As for many other hormonal factors, the level of circulating IGF-1 gradually declines with age [108]. Supporting its WM protective effect, a recent study involving more than 369,000 participants from the UK biobank has revealed that higher concentrations of circulating IGF-1 are associated with greater WM volumes and smaller WMH [109]. In addition to its function as a neurotrophic factor, IGF-1 also regulates vascular remodeling [110], indicating its potential contribution to the retention and improvement of cerebral blood flow [111, 112]. Therefore, maintaining IGF-1 levels by PE can also benefit CNS myelination via the first two mechanisms referenced above. Additionally, VEGF, a factor beneficial to CNS myelination through its potent pro-angiogenic effect, is secreted by blood vessel-associated cells in response to lactate released from exercising skeletal muscles [113].

The fourth mechanism relevant to the effects of PE is neuronal activity-dependent myelination. It is described as a physiological phenomenon by which axonal electric activity instructs OLs to specifically myelinate active axons, thereby increasing their conduction velocity [114]. This process was first suggested in the 1960s after observing that myelination of the optic nerve is delayed in animals grown in the dark during early postnatal development [115]. Since then, various *in vitro* and *in vivo* studies have been performed to further understand this phenomenon [116-119]. More recently, Gibson et al. have elegantly used optogenetics to drive motor cortical neuronal activity and to demonstrate that increased neuronal firing promotes circuit-specific OL lineage maturation and myelin formation [120]. The precise molecular mechanism remains to be elucidated; however, action potential-triggered leakage of components, such as potassium and glutamate, from unmyelinated axon sites, is considered to locally mediate a series of OL lineage responses promoting myelination [118, 121]. Beyond vitalizing motor control-related neural circuits, PE also stimulates many other neural networks associated with sensation, emotion and cognition. Therefore, PE can be expected to promote myelination at a wide range of CNS unmyelinated axon sites, including those involved in cognition.

The last anti-aging mechanism related to mental stress was not appreciated until the 21st century when researchers started to report OL and CNS myelin abnormalities in humans and animals under psychologically stressful conditions [122-126]. A likely mechanistic explanation is that cortisol, the hormone known to promptly increase in the blood in response to various stressors, decreases OL precursor proliferation and delays CNS myelination [127, 128]. Based on a systematic review and meta-analysis, Ownby et al. pointed out that individuals with a history of depression are more likely to be diagnosed with AD later in life [129]. Other cohort studies have found that late-life depression may even indicate the prodromal stage of AD [130, 131]. Importantly, depression due to persistent feelings of loneliness and health anxiety are commonly reported in elderly populations [132]. Altogether, while more work is needed to understand how mental stress exactly impairs OL functions, the well-recognized effect of PE on improving depressive symptoms [133, 134] can contribute to mitigating mental stress-associated WM damage, which could later cause AD.

## 2. Anti-AD effect

In addition to the general anti-brain aging effects of PE described above, several groups have reported some PE-driven benefits more directly relevant to the molecular and cellular mechanisms underlying demyelination in AD. First, a recent study in the APP/PS1 mouse model has reported that aerobic exercise, in the form of swimming (1 hour/day, 5 days/week, for 12 weeks), can restore the synthesis of the key myelin component, cholesterol, as well as its overall homeostasis that involves both glial and neuronal cells in the brain [135]. While the authors did not evaluate the impact of PE on myelination specifically, they demonstrated that regular exercise improves mouse cognition and activates the AKT pathway. Activation of the AKT pathway leads to the translocation of sterol regulatory element-binding protein 2 (SREBP2) to the nucleus and the ensuing coordinated induction of key cholesterol synthesis enzymes, including SEC24D which is selectively impaired in APP/PS1 mice. Consistent with our discussion in the previous section about the molecular and cellular mechanism of AD, normalization of local cholesterol synthesis and cellular transport could thus be a major mediator of the beneficial effects of PE on myelin maintenance and cognition preservation.

Another recent work with triple transgenic APP/PS1/tau mice focused specifically on the effect of PE on myelin alterations [95]. Qiu et al. showed that regular aerobic exercise regimen (motor-drive treadmill, 1 hour/day, 5 days/week, for 6 months) ameliorates not only AD-linked myelin damages but also impaired OL differentiation, which translated into improved learning and memory performance in the mice. Although the molecular mechanisms remained to be elucidated, these findings indicate that PE can restore normal OL maturation and differentiation that are major determinants, as we previously discussed, of myelin aberrations in AD. A study performed in a different rodent model of myelin lesion, such as hypoxic-ischemic brain injury in rats, may give us some molecular clues about the likely involved pathway [136]. Indeed, this work showed that constraint-induced movement therapy, comparable to PE in disabled animal models, activates the Sox2/Fyn pathway to overrun the RhoA/ROCK signaling and promotes OL maturation and differentiation. Incidentally, many studies have reported that the RhoA/ROCK signaling pathway can drive the development of several AD pathogenic hallmarks, including amyloid-beta and neurofibrillary tangle formation [137].

Finally, we want to highlight that PE also exerts some neuroprotective effects. This is important as functional myelin sheaths cannot be formed without healthy axons and neuronal bodies. Among PE-induced myokines, fractalkine was, for instance, reported to stimulate neurogenesis [138], while FGF2 was shown to repress amyloid-beta production and suppress neuronal death [139, 140]. BDNF and IGF-1, two pro-myelination factors discussed earlier, are also capable of reducing amyloid-beta accumulation and protecting neurons [141, 142]. In animal models of AD and aging, PE was also shown to mitigate CNS mitochondrial abnormalities, such as defective energy production and Drp1 hyperactivation, as we mentioned earlier [143-145]. A recent observation made in the D-galactose mouse model of accelerating aging and cognition decline is that PE (daily treadmill training protocol with increasing intensity over 8 weeks) can downregulate the pro-neurodegenerative microRNA, miR-34a [146]. In this study, decreasing the levels of miR-34a by PE led to the activation of the TAN1/PI3K/AKT/CREB signaling pathway, resulting in the promotion of myelin repair and neuroprotection.

Together, while the underpinnings of the beneficial effect of PE on apoE4-associated demyelination remains to be further clarified [147], the wide array of molecular targets that PE activates might contribute to moderating this pathology. One human study did report more declined WM integrity in physically active APOE4 carriers compared to less-active APOE4 carriers [148]. Thus, further studies are needed to tease apart this potential discrepancy. Nevertheless, given that AD is etiologically heterogeneous and involves spatiotemporally evolving and multifaceted pathophysiological processes, we emphasize that PE, which can simultaneously activate multiple mechanisms for CNS myelin repair, is particularly a well-suited AD modifying strategy (Fig. 2).

## 3. Optimal PE for AD

We have summarized the potential of PE to promote CNS myelination and thus serve as an intervention to delay progression of aging-associated WM demyelination, a step we underscore as essential in the development of AD. Yet, identifying the optimal PE form for moderating AD remains to be investigated. PE can be classified into open-skill exercise (OSE) and closed-skill exercise (CSE) as recently detailed by Yamasaki [17]. The former (e.g., table tennis, badminton, soccer, baseball, martial arts) is performed in dynamic, externally paced and unpredictable environment requiring active decision-making, whereas the latter (e.g., running, swimming, machine workout, gymnastics, cycling) is performed in relatively consistent, self-adjustable and predictable environment with less cognitive requirements [149]. The results of previous systematic reviews and meta-analyses suggest the greater benefit of OSE for improving cognitive functions of elderly people, presumably due to greater cognitive demands and social interaction of OSE versus CSE [150-152]. Consistently, Behrendt et al. reported that levels of beneficial factors promoted by exercise, such as BDNF and IGF-1, increased more in the blood of a group of older adults assigned to chronic OSE than the corresponding CSE group [149]. Despite the superiority of OSE, it must be noted that both types of PE have pro-cognitive effects. However, physical potential gradually declines with age and some older individuals often manifest limited mobility due to various morbidities or injuries, making it impractical to even carry out relatively easy-to-perform CSE. As an alternative for people with limited mobility, researchers are currently studying the application of electrical muscle stimulation as a means of maintaining muscle health and triggering pro-cognitive exercise factor release for the benefit of brain health [153, 154]. A conceptually similar but more direct approach of passive neuronal stimulation, including transcranial magnetic stimulation (TMS), is increasingly gaining attention as a novel technique potentially applicable for driving neuronal activity-dependent CNS repair in patients with neurological disorders. Indeed, several studies in animal models and patients with MS have shown TMS effectively enhancing CNS remyelination and moderating symptoms, as previously summarized by Maas and Angulo [155]. More recently, Zong et al. demonstrated, in a rat model of ischemic stroke, that application of continuous theta-burst stimulation, a specific modality of TMS, significantly upregulates neurotrophic factors, such as BDNF and FGF2, enhances neurogenesis and oligodendrogenesis, promotes myelination, and improves motor function [156]. Of note, TMS is already in clinical use for patients with treatment-resistant depression [157]. While further basic and clinical studies are needed, this technique may be applied in the near future to lessen WM demyelination and ensuing cognitive decline, in particular, in early AD patients who cannot practice regular PE due to limited mobility.

### Conclusion

Keeping an active daily life, both physically and intellectually, remains to be one of the few compelling strategies to slow aging-associated cognitive decline in elderly adults. As highlighted in this review, the latest evidence indicates that CNS myelin loss (i.e., WM demyelination) is (i) one of the earliest AD pathologies appearing preclinically, (ii) a major cause of amyloid-beta accumulation and (iii) potentially modifiable by PE through multiple mechanisms. Accordingly, we conclude that PE should counteract aging-associated WM demyelination causing cognitive decline in AD. More basic science and clinical studies are warranted to open new therapeutic avenues to prevent and slow AD progression, and to achieve healthier brain aging among the general population. Building a society where senior citizens can more easily and comfortably participate in PE should contribute to alleviating many public health issues in our increasingly aging global population.
